# Therapeutic Approaches Targeting the Natural Killer-Myeloid Cell Axis in the Tumor Microenvironment

**DOI:** 10.3389/fimmu.2021.633685

**Published:** 2021-04-19

**Authors:** Larissa S. Carnevalli, Hormas Ghadially, Simon T. Barry

**Affiliations:** Early Oncology, Research and Development, AstraZeneca, Cambridge, United Kingdom

**Keywords:** immunotherapy, cancer immunotherapy, myeloid cell, NK cell, tumor microenvironment

## Abstract

Immunotherapy has transformed cancer treatment by promoting durable clinical responses in a proportion of patients; however, treatment still fails in many patients. Innate immune cells play a key role in the response to immunotherapy. Crosstalk between innate and adaptive immune systems drives T-cell activation but also limits immunotherapy response, as myeloid cells are commonly associated with resistance. Hence, innate cells have both negative and positive effects within the tumor microenvironment (TME), and despite investment in early clinical trials targeting innate cells, they have seen limited success. Suppressive myeloid cells facilitate metastasis and immunotherapy resistance through TME remodeling and inhibition of adaptive immune cells. Natural killer (NK) cells, in contrast, secrete inflammatory cytokines and directly kill transformed cells, playing a key immunosurveillance role in early tumor development. Myeloid and NK cells show reciprocal crosstalk, influencing myeloid cell functional status or antigen presentation and NK effector function, respectively. Crosstalk between myeloid cells and the NK immune network in the TME is especially important in the context of therapeutic intervention. Here we discuss how myeloid and NK cell interactions shape anti-tumor responses by influencing an immunosuppressive TME and how this may influence outcomes of treatment strategies involving drugs that target myeloid and NK cells.

## Introduction

Immunotherapy has transformed cancer treatment by harnessing the immune system to target solid and hematological cancers (1), achieving durable responses across multiple tumor types (2). However, only approximately 20% of patients have a durable response, and intrinsic or acquired resistance is often observed in the clinic (3, 4). Therefore, novel combination approaches are needed to expand the therapeutic benefit of these drugs. Currently, several anticancer therapies employing multiple drug modalities and combinatorial approaches are being tested clinically (5), but few have been found to enhance benefit in combination with checkpoint inhibitors.

Because the balance of immunosuppressive versus immunostimulatory cells varies among individual tumors, a major goal of these approaches going forward is to understand immune contexture and segmentation. Most solid tumors include a variety of immunosuppressive cells, such as regulatory T cells, polymorphonuclear (PMN) myeloid-derived suppressor cells (MDSCs), mononuclear MDSCs (M-MDSCs), tumor-associated macrophages (TAMs) [as defined by Bronte et al., (6)] and neutrophils that can suppress effector CD8^+^ T cells and NK cells (7).

Two cell types of the innate immune system that shape the tumor microenvironment (TME) and can initiate anti-tumor immune responses are natural killer (NK) cells and cells of the myeloid lineage, including immunosuppressive PMN-MDSCs, M-MDSCs, and immune-activating macrophages, dendritic cells, and neutrophils. Although the individual roles of these cell types in the anti-tumor immune response have been extensively studied [for review, see Neophytou et al., (8)] the ways in which interactions between these cell types affect immune responses is only just emerging. Consideration of the interplay between NK cells and suppressive myeloid cells could give new insight into the effects of therapies combining PD-1/PD-L1 and CTLA-4 blockade in the clinic and may also have an impact in early-stage cancers and hematological diseases.

The most advanced therapies are those that modulate myeloid cells, depleting or inhibiting recruitment or promoting reprogramming to activate or de-repress tumoricidal mechanisms (9), but these modalities have been disappointing in the clinic (**Table 1**). These trials include inhibitors of CSF1R, CCR2, CXCR2, CXCR4, and most recently, PI3Kγ (10, 11). Although many of these modalities have been tested clinically, few have passed beyond phase 2 studies, due to either lack of efficacy or associated adverse effects. It is important to gain insight into the mechanism of action and biomarker changes associated with efficacy in order to refine therapeutic strategies for myeloid-targeting agents and to identify patients who could benefit from these therapies as monotherapies or in combination with immune checkpoint blockade.

**Table 1:** Myeloid and NK target therapies tested in clinical studies.

Target	Mechanism of Action	Modality	Drugs/Company	Dose regimen	Current clinical status	Combinations	Indications	Clinical trial number
CCR2	CCR2 is expressed by monocytes and macrophages and interacts with CCL2 to mediate chemotaxis of monocytes and TAMs, promoting tumor progression	Small molecule	PF-04136309 (Pfizer)	Continuous	Discontinued post-phase 1b/2	Folfirinox	Pancreatic ductal adenocarcinoma	NCT01413022
						Nab-paclitaxel		NCT02732938
CSF1R	CSF1 receptor (CSF1R)-mediated signaling is crucial for the differentiation, recruitment, and survival of the mononuclear phagocyte system and macrophages	Small molecule	Pexidartinib (Turalio) (PLX7486), Daiichi Sankyo	Continuous	Phase 2/approved	Monotherapy	Tenosynovial giant cell tumor	NCT01804530
		Small molecule	JNJ-40346527 (J&J)	21-day cycle or PO BID for 4–5 weeks	Discontinued after phase 1b/2	Monotherapy	Relapsed or refractory Hodgkin lymphoma	NCT01572519
							Relapsed or refractory AML	NCT03557970
						Surgery	Advanced Prostate Cancer	NCT03177460
		Small molecule	ARRY-382 (Array/Pfizer)	21-day treatment cycles	Phase1b	Keytruda (anti–PD-1 antibody)	Relapsed or refractory Hodgkin lymphoma, AML	NCT02880371
								NCT01316822
		Small molecule	BLZ945 (Novartis)		Phase I (ongoing)	PDR001 (anti–PD-1)	Advanced solid tumors	NCT02829723, NCT02404441
		Antibody	RG7155/emactuzumab (Roche)	IV Q3W	Phase 2	Atezolizumab (anti–PD-L1 mAb)	Advanced solid tumors	NCT02323191
						Selicrelumab (anti-CD40)		NCT02760797
						Paclitaxel and bevacizumab	Platinum-resistant ovarian cancer	NCT02923739
		Antibody	AMG 820-mAb (Amgen)	IV weekly	Phase 1/2	Pembrolizumab (anti–PD-1 mAb)	Advanced solid tumors	NCT02713529, NCT01444404
		Antibody (human mAb)	LY3022855 (Lilly)	IV Q4W	Phase 1	Durvalumab (anti–PD-L1 mAb) or tremelimumab (anti–CTLA-4 mAb)	Advanced solid tumors	NCT02718911
						GVAX		
							Pancreatic cancer	NCT03153410
CXCR2/IL8 axis	CXCR2 plays a critical role in the regulation of neutrophil homeostasis and recruitment to the tumor	Small molecule	AZD5069 (AstraZeneca)	Continuous + PD-L1	Phase1/2	Durvalumab (anti–PD-L1 mAb)	Head & neck/pancreatic cancer	NCT02499328, NCT02583477
						Enzalutamide	mCRPC	NCT03177187
		Antibody	HuMax-IL8/BMS-986253 (BMS)	IV Q2W	Phase1/2	Nivolumab + degarelix	Hormone-sensitive prostate cancer	NCT03689699
						Nivolumab		
							HCC	NCT04050462
							metastatic or unresectable solid tumors	NCT03400332
							NSCLC/HCC	NCT04123379
		Small molecule	Navarixin/MK-7123 (Merck)	IV infusion on day 1 of each 3-week cycle	Phase 2	Pembrolizumab	Advanced/metastatic solid tumors	NCT03473925
		Small molecule	SX-682 (Syntrix Pharmaceuticals)	SX-682 monotherapy for 21 days, then 90 days with pembro	Phase 1	Pembrolizumab	Metastatic melanoma	NCT03161431
			Reparixin (IL-8) (Dompe)		Phase 2; discontinued	Paclitaxel	HER2^–^ breast cancer	NCT02001974
								NCT02370238
		Small molecule						NCT01861054
PI3Kγ	PI3Kγ signaling promotes macrophage pro-inflammatory profile and anti-tumor activity	Small molecule	Eganelisib (IPI-549)	Continuous	Phase 2	Nivolumab	Advanced urothelial carcinoma	NCT03980041 UC
						Tecentriq and abraxane (TNBC)/bevacizumab (RCC)	TNBC and RCC	NCT03961698 RCC
						AB928 (A2ARi)/pegylated liposomal doxorubicin (PLD)/nanoparticle albumin-bound paclitaxel (NP)	TNBC and ovarian cancer	
								NCT03719326 TNBC/OV
								NCT03719326 TNBC/GC
CCL2	CCL2 chemokine interacts with CCR2 in monocytes and macrophages, impairing migration	Antibody (human mAb)	Carlumab (CNTO888)	IV Q2W	Phase 2	Monotherapy	MCRP	NCT00992186
						Chemotherapy (SoC)	advanced solid tumors	NCT01204996
CD47/CD47-SIRPα	Promotes the adaptive immune response and enhances the phagocytosis of tumor cells by macrophages	Antibody (hu mAb)	Magrolimab (Hu5F9-G4)/Gilead Sciences	IV every 3 cycles	Phase 3	Azacitidine	MDS	
							AML	NCT03248479
							DLBCL	
							FL	
		Antibody (hu mAb)	CC-90002/Celgene	IV infusion on a 28-day cycle	Phase 2	Rituximab	Advanced solid and hematologic cancers	DOI: 10.1056/NEJMoa1807315
								NCT02367196
NK2GA	NKG2A/CD94 are inhibitory receptors expressed on T and NK cells. Inhibition of interaction with HLA-E relieves inhibitory signals and leads to cell activation and cytotoxicity	Antibody (hu mAb)	Monalizumab	IV	Phase 1/2 Phase 3	Durvalumab (MEDI4736)	advanced solid tumors	NCT02671435
						Ibrutinib	Relapsed, refractory or previously untreated CLL	
						Durvalumab	Advanced NSCLC (resistance CPI)	NCT02557516
						Durvalumab	NSCLC	NCT03833440
						Durvalumab	Resectable NSCLC	NCT03822351
						Cetuximab	Metastatic HNSCC	NCT03794544
								NCT02643550
						Cetuximab	Recurrent or metastatic HNSCC	NCT04590963
CD30xCD16a	AFM13 is a bispecific, tetravalent chimeric antibody designed for the treatment of CD30-expressing malignancies. AFM13 recruits NK and macrophage cells *via* binding to CD16A as immune effector cells. https://dx.doi.org/10.1182%2Fblood-2014-12-614636	Affimed	AFM13	Weekly IV	Phase 2	Pembrolizumab	Relapsed or refractory classical Hodgkin lymphoma	NCT02665650
				Weekly IV	Phase 1/2 approved (orphan drug designation)		Peripheral T-cell lymphoma	NCT04101331
EGFRxCD16A	AFM24 NK-cell–engaging bispecific antibodies to target EGFR-expressing tumor cells irrespective of their mutational status.	Bispecific engager	Affimed (AFM24)	Weekly IV	Phase 1		Advanced solid cancers	NCT04259450
BCMAxCD16a	Bispecific antibody (IgG-scFv) targeting B-cell maturation antigen and CD16a (FcγRIIIA) being developed for treatment of multiple myeloma	Bispecific engager	Roche (RO7297089)	Weekly IV	Phase 1		Multiple myeloma	NCT04434469
HER2 x NKG2D x CD16A	HER2 trispecific NK cell engager; binds to HER2 on tumor cells and simultaneously binds to NK cells	Trispecific engager	Dragonfly Therapeutics (DF1001)		Phase 1/2	Pembrolizumab	Advanced solid tumors	NCT04143711
KIR2DL-1, -2, -3	Inhibits major inhibitory receptors on NK cells	Humanized mAb	Innate Pharma/BMS (IPH2102/BMS-986015/lirilumab)	4 cycles Q4W IV	Phase 1/2	Ipilimumab or nivolumab	Advanced solid tumors	NCT01750580 NCT01714739
CD16/IL-15/CD33	Trispecific scFv recombinant fusion protein conjugate composed of heavy and light chains of anti-CD16 and anti-CD33 antibodies and human IL-15	Trispecific engager	GT Biopharma (GTB-3550)	3x weekly IV	Phase 1/2		High-risk heme malignancies	NCT03214666

AML, acute myeloid leukemia; BID, twice daily; CLL, chronic lymphocytic leukemia; CPI, checkpoint inhibitor; EGFR, epithelial growth factor receptor; HCC, hepatocellular carcinoma; HNSCC, head and neck squamous cell carcinoma; IV, intravenous; mAb, monoclonal antibody; m-CRPC, metastatic castration-resistant prostate cancer; MDS, myelodysplastic syndrome; NP, nonpegylated; NSCLC, non–small-cell lung carcinoma; PLD, pegylated liposomal doxorubicin; PO, orally; PTCL, peripheral T-cell lymphoma; Q2W, Q3W, Q4W, every 2, 3, 4 weeks; RCC, renal cell carcinoma; scFv, single-chain variable fragment; SoC, standard of care; TNBC, triple-negative breast cancer.

n contrast, only a few drugs targeting NK biology to reverse NK tumor immune tolerance have been progressed to clinical trials (**Table 1**). These therapies include the anti–KIR2DL-1, -2, and -3 antibody IPH2102/BMS-986015 (lirilumab), the anti-NKG2A antibody IPH2201 (monalizumab), and the anti-CD16 innate cell engager AFM13.

To date, the concept of modulating NK-myeloid cell interactions to relieve tumor immunosuppression is underexplored. However, further consideration of NK-myeloid cell interactions in the TME and periphery may provide insights into both innate and adaptive immune anti-tumor responses. Here we discuss possible mechanisms that can attenuate or enhance a productive immune response through innate cell–mediated responses and the consequence for activation of effector cell types in the TME.

## NK Cell Biology and the TME

NK cells are large, granular lymphocytes that can kill target cells without previously encountering an antigen. NK cells also produce proinflammatory cytokines like interferon-alpha (IFN-α), tumor necrosis factor-alpha (TNFα), and granulocyte macrophage–colony-stimulating factor (GM-CSF), as well as chemokines such as CCL1, CCL3, CCL4, CCL5, CCL22, and CXCL8. Their activity is regulated by a balance of signals from activating and inhibitory receptors (12). Most of the inhibitory receptors bind to major histocompatibility complex (MHC) class I–like proteins, which enable NK cells to detect the downregulation of MHC class I molecules on target cells. Activating receptors, on the other hand, bind a variety of molecules, some of which are derived from pathogens such as CMV protein pp65, which is recognized by NKp30 (13), or are induced by cell stress, transformation, or infection (e.g., MICA/B and ULBP1-6, the ligands of NKG2D) (14). NK cells not only play an important role as a first line of defense against viral, bacterial, and fungal infections (15, 16), but are also important in tumor immuno-editing (17), tumor development (18), and control of metastasis (19–21).

Under nonpathological conditions, NK cells and myeloid cell subtypes crosstalk through multiple mechanisms. NK cells interact with macrophages and dendritic cells through both soluble factors, such as IL-12, IL-15, IL-27, and IL-18, and cell-to-cell contact (22–24). These interactions can induce maturation of NK cells, cytotoxicity, and cytokine release. Reciprocally, NK cell–derived cytokines can drive stimulation of macrophages. Pathogen-induced upregulation of ligands for activating NK-cell receptors can result in the elimination of monocytes and macrophages by NK cells (25), as well as the killing of immature but not mature dendritic cells *in vitro* (26), a process thought to limit the generation of potentially tolerogenic dendritic cells.

## Myeloid Cells in the TME Influence NK Function

Tumor-derived myeloid cells are plastic and heterogeneous and have both positive and negative roles in anti-tumor immunity. There are two main subsets of suppressive myeloid cells in tumors, PMN-MDSCs and M-MDSCs (6, 27, 28). Monocytes, M-MDSCs, and TAMs are abundant in solid tumors (29) and are associated with poor prognosis (30, 31). M-MDSCs support tumor progression through both immune-mediated mechanisms and mechanisms not directly associated with immune suppression (32). Macrophages and monocytic MDSCs isolated from mouse murine and human solid tumors can directly suppress T‐cell responses (29, 33) and NK-cell cytotoxicity (34). M-MDSCs are implicated in the recruitment of T regulatory cells and inhibition of T-cell cytotoxicity and have been shown to inhibit NK cell function *in vitro* and *in vivo* (35). Normally, neutrophils respond to tissue damage and defend against pathogens (36), but in the TME, tumor-associated neutrophils or PMN-MDSCs express various cytokines, including CCL2 and CCL17, depending on their immunosuppressive or immune-activating state, and can degranulate to release various types of bioactive molecules (37, 38). The formation of neutrophil extracellular traps can convert dormant cancer cells, drive aggressive lung metastases in mice (39), and accelerate hepatocellular cancer (40) in patients and in mouse models (41).

## NK Cells in the TME

The TME not only shapes the adaptive immune response but also has profound effects on NK cells, which in many tumors are functionally distinct. Anti-tumor NK effector mechanisms such as cytotoxicity and secretion of pro-inflammatory cytokines are impaired due to low expression of effector molecules perforin and granzyme in patients with lung adenocarcinoma (42), downmodulation of activating receptors NKG2D or NKp30 in gastric cancer (43), and upregulation of inhibitory receptors like NKG2A in cervical cancer (44).

NK cells in tumors also acquire pro-angiogenic and pro-tumor functions, including the secretion of vascular endothelial growth factor (VEGF) (45), angiogenin, and MMP9 (46, 47). Indeed, NK cells play an important role in the menstrual cycle and establishing the placenta (48). The induction of some of these phenotypic features have been attributed to immune-modulatory molecules present in the TME, such as indoleamine-pyrrole 2,3-dioxygenase and tumor growth factor-beta (TGF-β), which can be secreted by MDSCs (49). NK cells in which STAT5 has been silenced express VEGF-A at a level sufficient to promote the growth of murine syngeneic tumors (50). NK cells with a pro-angiogenic phenotype have been identified in non–small-cell lung cancer (47) and colorectal cancer (51). However, it is not clear whether there is a meaningful or broad contribution of these potentially pro-angiogenic NK cells to drive tumor progression or whether they represent the primary angiogenic drive. In one study, genetic inactivation of VEGF in myeloid cells prevented tumor growth and chemotherapy-induced cachexia in B16 and LLC mouse tumor models (52). This study also suggested that increased levels of circulating chemerin by the tumor endothelium improved NK-cell recruitment to the tumor site, suggesting that an indirect mechanism of targeting myeloid cells affects NK recruitment and function. It would be important to understand whether pharmacological interventions would have a similar effect and whether this is a dominant mechanism.

Some of the factors that are known to contribute to functional impairment of NK cells, such as hypoxia, are tumor intrinsic, whereas others are secreted by tumor-associated cells, in particular MDSCs and TAMs. In mouse models, one such mechanism is induction of NK-cell scavenger receptor expression, which is involved in lipid metabolism. Uptake of MDSC-derived factors leads to lipid accumulation and functional impairment (53).

NK cells have also been implicated in anti-tumor immune responses after checkpoint blockade. PD-1 is expressed on about 25% of NK cells in some healthy donors, usually at low levels (54), but has been found to be expressed at substantial levels in patients with ovarian cancer (54); digestive cancers, including esophageal, liver, colorectal, and gastric cancers (55); multiple myeloma (56); Kaposi sarcoma (57); and renal cell carcinoma (58). However, infiltrating NK cells in non–small-cell lung cancer do not express PD-1 (59), although a recent systematic study using multiple methods to detect PD-1 protein and mRNA concluded that NK cells showed only minimal expression of PD-1 in primary human tumor samples of round-cell sarcoma and colorectal cancer, as well as in multiple mouse tumor models (60). Despite these findings, several studies have reported upregulation of PD-1 expression on NK cells in various mouse models (61, 62), and although blockade of PD-1/PD-L1 interaction has been shown to enhance activity of NK cells *in vitro* and in animal models (63), this is suggested to be mediated mainly through expression of PD-L1 by NK cells (64). Moreover, it is not clear how NK cells contribute to anti-tumor responses in patients. NK cells have also been implicated in playing a role in response to treatment with agonistic anti-CD137/4-1BB antibodies. CD137 is upregulated by Fc receptor cross-linking on NK cells (65) and in patients after treatment with monoclonal antibodies (66). CD137 ligation contributes to activation *in vitro* (67) and in humanized mice (68) but reports that enhances antibody-dependent cell cytotoxicity have been retracted (69, 70).

## Humoral vs. Cell-Cell Interaction Crosstalk Between NK and Myeloid Cells

In the TME, cell-cell interactions and humoral responses build an anti-tumor immune response; therefore, it is important to consider how different therapeutic approaches can affect these interactions. The role of myeloid cells in the TME has been extensively studied, and a number of therapeutics have been developed to target these cells. Both neutrophil/PMN-MDSC and macrophage/M-MDSC–like myeloid cells can influence both T-cell and NK-cell activation and play both positive and negative roles in tumor growth and metastatic progression.

**Figure 1:**
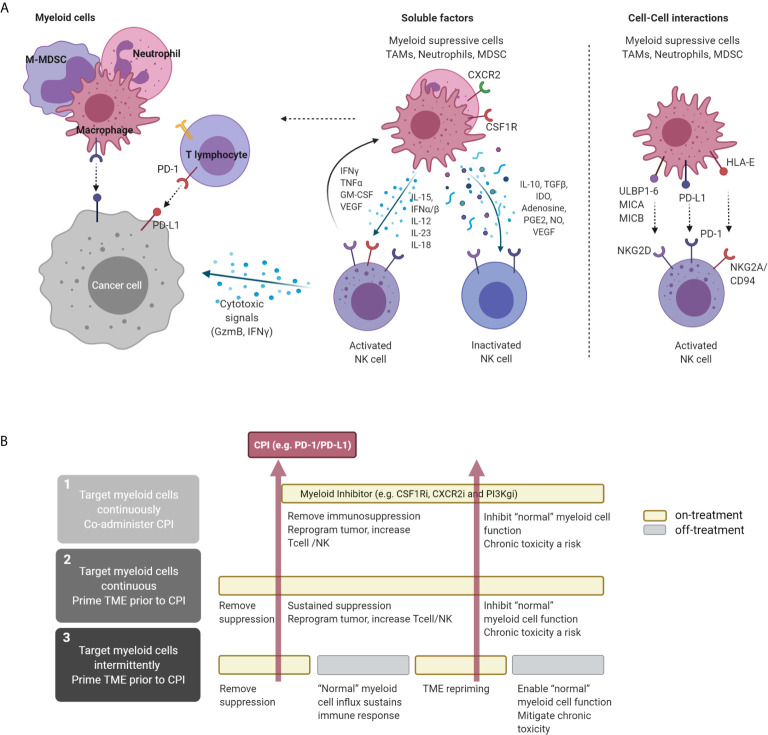
Direct and indirect interaction of NK and myeloid cells in the TME and therapeutic concepts. **(A)** Interaction of NK and myeloid cells in the TME. NK cells can directly target tumor cells *via* cytolytic granules, independent of antigen recognition. Macrophages with immunostimulatory properties can independently induce tumor-cell killing through antigen presentation and production of pro-inflammatory cytokines. In the TME, myeloid cells, including TAMs, M-MDSCs, neutrophils, and PMN-MDSCs, can secrete a variety of soluble factors that inhibit NK activation and therefore suppress NK-mediated cytotoxicity. Cytokines secreted by NK cells (e.g., IFNγ, TNFα, and GM-CSF) can stimulate macrophages, driving a pro-inflammatory activated state. These two cell types can also interact at the receptor level, where myeloid cell-surface ligand and NK receptors interact, attenuating downstream signaling, e.g., NKG2D. **(B)** Proposed therapeutic approaches targeting myeloid-cell subsets in the TME and proposed alternative treatment sequences that can be explored to maximize immune-mediated anti-tumor response. CPI, checkpoint inhibitor. Figure created with BioRender.com.

During infection, macrophages can modulate NK function either through direct cell-to-cell contact or through secretion of soluble mediators such as IL-18, IL-12, and TGF-β (25) (**Figure 1A**). CD56^bright^ NK cells accumulate in inflammatory lesions in the presence of IL-12, IL-15, and IL-18 and engage with CD14^+^ monocytes in a reciprocal activation loop, amplifying the inflammatory response by increasing TNFα production by monocytes and IFNγ by NK cells (71). *In vitro*, appropriately activated myeloid cells can also facilitate activation of NK cells *via* cell-cell interactions, enhancing CD69 expression and secretion of IFNγ in co-cultures (71, 72). In contrast, monocytes and macrophages isolated from hepatocellular carcinoma patient samples (34) and gastric cancer (73) tumors can induce NK-cell dysfunction *via* direct cell-cell interaction and indirectly, e.g., through soluble TGF-β signaling. In other studies, macrophages and monocytes isolated from hepatocellular carcinoma samples expressed high levels of CD48, driving NK-cell dysfunction. This effect was attenuated by blocking the NK-cell CD48 receptor 2B4 (34). Macrophage or M-MDSC secreted factors can have direct and indirect effects on myeloid and NK-cell crosstalk. Soluble TGFβ modulates NK-cell function *via* activating receptors NKG2D and CD16 antibody-dependent cell-mediated cytotoxicity in tumors by impairing cytotoxicity potential *in vivo* and in co-culture experiments with acute myeloid leukemia and colon cancer models (74, 75). Conversely, IL-15 plays a role in maintaining NK activation to suppress tumor escape and metastasis (76). Other secreted factors may act indirectly; these include tumor-derived prostaglandin-E2, which induces MDSCs and inhibits NK-cell function in melanoma samples (77). Restoring NK-cell function by co-targeting immunosuppressive myeloid cells may be an important therapeutic strategy to prevent tumor immune escape (**Figure 1A**).

Pro-inflammatory macrophages, such as IL-12–secreting macrophages (9, 78), that promote NK function in infection and mouse tumor models highlight the importance of understanding the difference between specific myeloid phenotypes and their influence on NK activation and function (79). Some myeloid-targeting therapies rely on cell depletion mechanisms, whereas others attempt to block recruitment or to reprogram these cells into a pro-inflammatory anti-tumor state (9). This is an important consideration when developing therapies, given the high plasticity of myeloid cell types and multiple cell interactions, including activating and suppressive impacts on T- and NK-cell effector functions.

Most studies have focused on the effects of myeloid-cell inhibitors (e.g., CSF1R inhibitors) on primary tumors, but not much information is available in the context of metastasis. CSF1R inhibition reprograms the TME to increase responses to chemotherapy and checkpoint inhibitors and to decrease metastatic spread (80). In some studies, inhibition of CSF1R depleted tumor-associated macrophages but unexpectedly promoted metastasis in 4T1 orthotopic syngeneic models. In one report, CSF1R inhibition reduced the number of NK cells due to a decrease in IL-15, a T-cell and NK-cell survival factor secreted by myeloid cells (22). Moreover, dosing exogenous IL-15 during CSF1Ri treatment restored NK-cell numbers and metastasis control. Genetic ablation of IL-15 in mice and in Th2-polarized CD4 T cells has been found to promote the formation of M2 macrophages that are thought to contribute to metastasis formation (81). NK cells may control the seeding of circulating tumor cells due to crosstalk with myeloid cells, a process that is affected when tumors are treated with depleting CSF1R antibodies (82). It is interesting to contrast this finding with the observation that neutrophils or PMN-MDSCs promoted metastasis in this model (83). Other therapeutic approaches can influence this axis. Blocking CD39 activity in myeloid cells has been shown to improve control of metastases *via* NK-cell effector function (84). CD39 expression by myeloid cells, but not NK cells, was required for efficacy, suggesting that blockade of CD39 on myeloid cells limits the impact of eATP in driving intratumor myeloid pyroptosis or the release of IL-18, both of which have been shown to stimulate NK-cell effector function (84).

CCL2 (MCP1), which interacts with CCR2, is an alternative mechanism that influences macrophage-related myeloid recruitment to tumors and subsequent tumor progression (85). Inhibition of CCL2 has been shown to limit early metastatic processes in breast cancer; however, after cessation of therapy, increased metastatic spread is observed due to enhanced recruitment of monocytes to micrometastatic lesions in breast (86, 87) and lung (88, 89) metastasis mouse tumor models. Importantly, it has been suggested that combined inhibition of CCL2 and IL-6, a cytokine expressed by myeloid cells, reduced metastasis and improved survival in prostate cancer (90). Targeting CCR2 also reduces tumor progression associated with an influx of T cells in preclinical glioma (91) and pancreatic models (92).

Although macrophages can contribute to anti-tumor immunity, both monocyte-derived MDSCs and TAMs can also promote cancer initiation, stimulate angiogenesis, and suppress anti-tumor immunity during malignant progression. Pro-inflammatory, or “anti-tumor”, macrophages contribute to an anti-tumor response by producing pro-inflammatory cues such as IFNγ and IL-12 secretion or by acquiring an antimicrobial and tumoricidal phenotype (93, 94). Therefore, “reprogramming” macrophages into an anti-tumor and proinflammatory state is an attractive strategy to tip the balance on tumor immunity. Targeting STAT3 (95) or PI3Kγ signaling has been shown preclinically to change the TME in tumors by remodeling suppressive macrophages into proinflammatory macrophages. Selective targeting of PI3Kγ signaling in combination with checkpoint blockade is thought to promote reprogramming of macrophages into a pro-inflammatory state, leading to cytotoxic T-cell–mediated anti-tumor response in preclinical mouse models (96, 97). The combination of PI3Kγ with PD-1 blockade is currently under investigation in clinical trials and recently received FDA Fast Track designation in urothelial cancers (ClinicalTrials.gov NCT03980041).

In normal physiological processes, neutrophil depletion impairs NK-cell maturation, function, and homeostasis (98). The role of neutrophils and PMN-MDSCs in cancer has been extensively studied, and these cells play an important role in facilitating tumor progression. In various tumor models, targeting or depleting neutrophils or PMN-MDSCs reduces metastasis in both autochthonous models of pancreas (99), colon cancer (100), breast (101), and metastatic syngeneic models (83, 102). This metastatic process may be through γδT-cell–orchestrated suppression of CD8 T cells by modified neutrophils (101). However, there is evidence that immunosurveillance of metastatic 4T1 cells by NK cells is inhibited by interaction with CD11B^+^/Ly6G^+^ neutrophils (most likely PMN-MDSCs), increasing residence time for metastatic tumor cells arriving at the lung and enabling extravasation and establishment of the metastatic niche (83). Soluble factors such as IL-17, granulocyte-CSF (G-CSF) (101), and TGFβ signaling (100, 103) play pivotal roles in establishing this suppressive network. The crosstalk between neutrophils and PMN-MDSCs is not a one-way process. In MCA205-Luc2 tumors, depletion of NK cells with antibodies or CXCR3 blockade has been shown to promote tumor growth due to reduced IFNγ and upregulation of IL-17A and VEGF-A, modifying the TME and recruitment of suppressive neutrophils of PMN-MDSCs (104).

Therapeutic targeting of CXCR2 (or IL-8) inhibits neutrophil-granulocytic myeloid cells or PMN-MDSCs, leading to suppression of metastasis in mouse models of pancreatic cancer (99) and colorectal cancer (100), as well as in metastatic syngeneic models 4T1 and B16F10 (105). In preclinical efficacy studies, CXCR2 inhibition resulted in an influx of T cells (99, 100, 105, 106); however, the impact on the broader immune environment, including NK biology, has not been explored. Although CXCR2 blockade inhibits recruitment of granulocytic myeloid cells to the tumor, it may also inhibit NK recruitment. CXCR1 and CXCR2 are highly expressed by cytotoxic CD56^dim^ NK cells (107), and increasing CXCR2 expression on NK cells promotes recruitment to tumors that overexpress CXCR2 ligands (108). Importantly, the CXCR2 ligand CXCL8 is secreted within the TME of melanoma-infiltrated lymph nodes and may play a role in the efficient recruitment of highly cytotoxic NK cells (109). Because it has been suggested that chronic combined inhibition of both CXCR2 and CSF1R can increase the efficacy of checkpoint inhibition in syngeneic models (110), understanding the potential impact of comprehensive myeloid suppressor cell inhibition on NK-cell activity should be considered.

Taken together, these studies of different myeloid lineages exemplify how depletion of specific subsets of myeloid cells can affect different features of the TME, modulating innate effector-cell activity and promoting tumor progression and metastasis. However, because myeloid cells, and particularly macrophages, play an important role in assisting NK- and T-cell activation, it is important to target the right population of cells. Moreover, given that these are essential cell types, translation to a clinical setting may be limited by tolerability, as observed in studies targeting the CSFR1 axis with antibodies or small molecules (80, 111, 112), which resulted in increased liver enzymes and induction of periorbital edema. Less toxicity was observed when the alternate macrophage regulating receptor CCR2 was targeted (113–115).

Nontargeted therapies, such as chemotherapy, can also deplete myeloid cells from tumor. Paclitaxel-carboplatin treatment was shown to alter circulating and intratumoral myeloid cell populations and to promote anti-tumor responses when combined with vaccination in HPV-16–positive tumors in mice (98). In a phase 2 trial in patients with extensive small-cell lung cancer, it was reported that ipilimumab treatment beginning with the third cycle of paclitaxel-carboplatin treatment produced better clinical outcomes than giving the drugs during cycles 1 to 4 (116). An understanding of the pivotal points in these complex signaling and transcriptional networks that program the myeloid cell phenotypes is essential to guide more effective therapeutic approaches.

## Perspective: Impact of Dose and Schedule in Myeloid Target Therapies and Checkpoint Inhibitors

Translation to the clinic of preclinical concepts, which were largely developed using fast-growing subcutaneous *in vivo* models, presents a challenge. Subcutaneous models are limited because they do not reflect the variations observed in the tissue of residence, and the speed of cell growth in these models does not enable elucidation of the longer-term consequences of the treatment strategy. As shown in **Table 1**, most clinical studies have taken a standard approach in which the myeloid therapy is co-administered with the checkpoint inhibitor or chemotherapy and then dosing is maintained chronically (80, 111–113, 115). This approach has a number of drawbacks. Myeloid cells exhibit both positive and negative effects on the TME, as described above. Accumulation of myeloid cells in the tumor (macrophage-like and neutrophil-like cells) clearly defines a resistance phenotype, and depletion of macrophages and neutrophils can remodel the TME. In addition to preventing the suppressive crosstalk to immune cell types, including T cells and NK cells, removal of these cells results in remodeling of the stroma and, in some cases, reprogramming of the tumor cell compartment. These changes make the tumor more susceptible to appropriate recruitment of activated immune cells. Hence, it is likely that pretreatment with a myeloid modulation agent prior to treatment with immunotherapy or even chemotherapy would “prime” the TME by reversing the resistant features in the tumor, facilitating more effective stimulation of the immune system. However, once the immune response is progressing, then more “normal” myeloid cells could be required to sustain that response, especially in situations where there is less effective immune recognition of the tumor. Paradoxically, chronic suppression of the myeloid cells may result in attenuation of the immune response in certain situations, mitigating the advantages gained from targeting the suppressive cells. Therefore, therapies that deplete myeloid cells or prevent recruitment to the TME may be more effective with intermittent or sequenced dosing, using the myeloid therapy for a short time prior to treatment to “prime” the TME, but then stopping dosing after introduction of the checkpoint inhibitor to allow the more normal immune response to progress (**Figure 1B**). These types of intermittent approaches could also mitigate clinical toxicity.

## Discussion

The development of cancer immunotherapies, specifically immune checkpoint blockade, has shifted the treatment of cancer by promoting complete and durable responses (117, 118). Immunotherapies focus on enhancing the activities of T cells; however, the complexity of the TME limits the response. The pivotal role of tumor myeloid cells, particularly macrophages, in conditioning the TME and regulating the broader response to host immune response and therapy is broadly appreciated. Unfortunately, the development of targeted therapeutics has only just started to teach us about the complexity of this cross-regulation, particularly in the context of different tumor mutational backgrounds and TMEs, as well as the broader systemic immune response. To enhance success, it is worth considering the positive influence of myeloid cells on the other components of the immune system, such as NK cells, and their role in sustaining persistent T-cell responses. Although myeloid therapies have largely been combined with checkpoint inhibitors and, to a lesser extent, chemotherapeutics, little consideration has been given to combinations with therapies targeting other functional nodes, such as NK cells or stimulators of innate immunity. As we seek to improve responses in patients earlier in disease progression, at the point of metastatic spread, such alternative strategies could become important.

## Author Contributions

All authors contributed to the article and approved the submitted version.

## Conflict of Interest

Authors are AstraZeneca Plc employees and shareholders.
